# Decision and coordination of low-carbon supply chain considering technological spillover and environmental awareness

**DOI:** 10.1038/s41598-017-03270-2

**Published:** 2017-06-08

**Authors:** Lang Xu, Chuanxu Wang, Hui Li

**Affiliations:** School of Economics and Management, Shanghai Maritime University, Shanghai, 201306 China

## Abstract

We focus on the impacts of technological spillovers and environmental awareness in a two-echelon supply chain with one-single supplier and one-single manufacturer to reduce carbon emission. In this supply chain, carbon abatement investment becomes one of key factors of cutting costs and improving profits, which is reducing production costs in the components and products—the investment from players in supply chain. On the basis of optimality theory, the centralized and decentralized models are respectively established to investigate the optimal decisions and profits. Further, setting the players’ profits of the decentralized scenario as the disagreement points, we propose a bargaining-coordination contract through revenue-cost sharing to enhance the performance. Finally, by theoretical comparison and numerical analysis, the results show that: (i) The optimal profits of players and supply chain improve as technological spillovers and environmental awareness increase, and the profits of them in the bargaining-coordination contract are higher than that in the decentralized scenario; (ii) Technological spillovers between the players amplify the impact of “free-ride” behavior, in which the supplier always incentives the manufacturer to improve carbon emission intensity, but the cooperation will achieves and the profits will improve only when technological spillovers and environmental awareness are great; (iii) The contract can effectively achieve coordinated supply chain, and improve carbon abatement investment.

## Introduction

From “United Nations Framework Convention on Climate Change” to “Kyoto Protocol”, and to “Copenhagen conference”, the voice of protecting environment is increasing, while the rapid growth of the world economy and continuous adjustments of economic structures are increasingly evident environmental pollution and resource shortages^1^. In order to achieve coordinated development of economy, resources and environment, the development of energy savings and reducing carbon emissions (ESER) can accelerate the construction of energy-saving and environmental protection standards, meanwhile, labeling and certification system can establish strict low-carbon products^2^. At present, carbon emissions reduction is regulated by the relevant government, but the essential parts of it still belongs to the external governance, the fundamental solution to reduce carbon emission is the investment of environment, especially the suppliers and manufacturers in the upstream and the downstream of supply chain. Carbon abatement investment for the suppliers is to ensure the availability of low-carbon manufacturers using raw materials and low-carbon customers using products.

As we known, carbon emission is believed to be the principal cause of global warming. Therefore, low-carbon supply chain has been has been a popular research topic in recent year, not only because of its great academic potential, also due to the increasing environmental stress on economic development. Under this background, carbon emission control drives enterprises to upgrade and modernize from the aspect of technology, but also transform their operation strategy from the aspect of management. In fact, existing products are gradually replaced with low-carbon products. With U.K. who has strong emission reduction awareness, for example, a survey result in end 2008 showed that over two-thirds people will give preference to purchase the products from the enterprises who take an active part in energy conservation and emission reduction^3^, which results in a tendency that high-carbon materials or products are gradually replaced with low-carbon materials or products. In other words, low carbonization drives enterprises to reconsider the operational and decision-making matters in their supply chain management. Therefore, the product substitutability aroused by low carbonization is bound to affect the supply chain management of enterprises significantly.

Further, since the 1990s, industrial organization theory is a very active area, which also affects the degree of competition and cooperation in technological innovation from the aspects of market equilibrium and social welfare^4^. These studies have tried to analyze the impact of technology innovation on supply chain decisions, investigate whether the impact of technology innovation on the competitors is positive or negative, and analyze the effect of technology innovation under different products and market. Under low-carbon economy, carbon emissions reduction cooperation between enterprises is beneficial to technology sharing among enterprises, speeding up technical innovation, and improving the competitiveness of supply chain^5^. The increasingly fierce competition and resource constraints in production strengthen the cooperation between the upstream and downstream enterprises and make the supply chain to form the vertical partnership^6^.

The innovation can promote players in the supply chain reducing the production cost and enhance the competitive advantage. However, R&D is likely to cause the effect of technological spillover thus weakening their competitive advantage^7^. The technological spillovers referring to the diffusion of technology through the promotion of technology to increase the level of productivity of other enterprises is a manifestation of economic externalities, mainly due to the release of technical information, or with other companies for technical exchanges. The effect of technological spillovers on the innovation decisions are as the following two aspects. On the one hand, due to the reduction of production cost, the firms need to develop their own technology to improve the research ability^8^; on the other hand, the firms can be free to share the technology from the other firms’ innovation with the effect of spillovers, which may damage their enthusiasm of innovation^9^.

Based on the above descriptions, our research shares several motivations are different from the previous research. We assume that the supplier supplies components to the manufacturer as a leader and the manufacturer sells products in the market as a follower. Similarly, the supplier absolutely controls the supply chain and is responsible for the channel of components to the manufacturer. Thus, in this paper, the following questions will be answered: 1. What is the impact of the relevant coefficients (eg. technological spillovers, environmental awareness and reduction difficulty coefficient) on the key decisions and profits of low-carbon supply chain players? 2. How does the bargaining-coordination contract through revenue-cost sharing between players in low-carbon supply chain impact the results? With these questions, the decisions and coordination of low-carbon supply chain have been studied in our paper.

### Literature review

There are main streams of literatures related to the topic of this paper: the first is the strategy of investment as the spillovers change, the second concern is the management of low-carbon supply chain, and the third explores the design of supply chain coordination.

First, many researchers have focused on the decisions of investment as the spillover changes. D’Aspremont *et al*. (1988) established a two-stage game model with the decisions of investment and R&D and compare the equilibrium of decentralized and centralized supply chain^10^. Jasper *et al*. (2015) found the duopoly market appear more cooperative and weaker competitive in the presence of the high spillover effect^7^. Chalioti *et al*. (2015) proposed supply chain R&D incentives principal model in the presence of technological spillover, found that technological spillover between enterprises and R&D cooperation are conducive to increase profits^11^. Wang *et al*. (2014) discussed the potential impact of spillover on the manufacturer’s incentives, proposed the method for improving the reliability of the supply chain cooperation^12^. Dietmar *et al*. (1996) developed a model in which a monopolist supplier can improve the downstream product by knowledge spillovers which the downstream enterprise uses as a substitute for their own R&D efforts^13^. Jeroen *et al*. (1990) compared the rationality of R&D under multidimensional game perspective, and takes into account the impact of spillover and stability^14^. Kotaro *et al*. (1992) introduced a suboptimal function the standard of the welfare to verify the positive correlation between R&D cooperation and the profit of entire supply chain^15^. However, the existing literatures only considered the horizontal technological spillovers, without analyzing the influence of the players in supply chain and technological spillovers effect on the decisions and profits.

In fact, regarding with the carbon emission reduction and carbon abatement investment in supply chain management, some scholars have recently issued the topic from the perspective of game theory. Ghost *et al*. (2012) built game theoretic models and show how greening levels, prices and profits are influenced by channel structures^16^. Cao *et al*. (2013) discussed the decisions of green supply chain based on Stackelberg game, and proposed the nonlinear pricing strategy through Nash bargaining^17^. Abdallah *et al*. (2012) developed a carbon-sensitive supply chain that minimizes emissions throughout the supply chain by taking into consideration green procurement^18^. Kim (2000) proposed an innovative subsidy from the manufacturer, promoting the supplier’s innovation and achieving the profit of supply chain^19^. Obviously, the above articles didn’t involve to how carbon abatement investment affects the production cost of the supply chain.

Moreover, the coordination of supply chain system has become popular. Banerjee *et al*. (2001) studied the vertical competing-cooperating model among upstream and downstream enterprises, and calculated the allocated incremental profits from total investment^20^. Kamien *et al*. (2000) provided three different cost-sharing contracts of cooperation, including income proportional sharing, production proportional sharing and fixed proportional sharing^21^. Ghosh *et al*. (2015) explored supply chain coordination issues arising out of green supply chain initiatives and explore the impact of cost sharing contract on the key decisions of supply chain players undertaking green initiatives^22^. John *et al*. (1972) extended Nash’s theory of two-person bargaining games with fixed threats to bargaining situations with incomplete information^23^. As is known, relevant studies on the coordination of supply chain are still at an early stage, therefore lack of research literature conducting quantitative analysis on specific problems.

In summary, all literature consider either horizontal technological spillover, or the demand of price-sensitivity. Our research intends to fill the gap in the previous literature by sharing a similar motivation, but comprehensively consider two dimensions—the technological spillover and environmental awareness in the decision and coordination of low-carbon supply chain. Then, the relationship between players’ decisions and the coordination contract has been analyzed. Therefore, it has great applicable meaning to the decision and coordination of low-carbon supply chain considering technological spillovers and environmental awareness which affect the decision and coordination of supply chain.

### Problem assumption and models

In this section, we first discuss the modeling assumptions, and the related decision results for the different scenarios—the centralized and decentralized where the decisions of optimal pricing and carbon emissions intensity for the supplier and manufacturer are analyzed. For ease of reference, the relevant coefficients of models are presented in Table 1.

**Table 1:** Definitions of coefficients.

*α*	The market potential demand
*γ*	The sensitivity coefficient of demand on the carbon emission intensity
*μ*	The difference between carbon emission intensity for the supplier and manufacturer on demand
*θ*_1_	The positive technological spillover that the supplier receives from manufacturer’s carbon emissions intensity
*θ*_2_	The positive technological spillover that the manufacturer receives from supplier’s carbon emissions intensity
*k*	The reduction difficulty coefficient in component and product
*w*	Decision variable, the price of unit component
*p*	Decision variable, the price of unit product
*e*_1_	Decision variable, carbon emissions intensity for the supplier
*e*_2_	Decision variable, carbon emissions intensity for the manufacturer

The basic assumptions are also needed throughout in our paper, as following:

**Assumption 1**

The two-echelon supply chain consists of one-single supplier and one-single manufacturer, in which the supplier and the manufacturer are respectively the leader and follower in the Stackelberg game. The supplier is a monopolist to the manufacturer, who is a monopolist to consumers. The supplier and the manufacturer are rational, and under the symmetric information with the technological spillovers, therefore *p* > *w* > 0.

**Assumption 2**

The manufacturer in the region forms a complete monopoly of the market, and the market demand faced by members in supply chain is a linear function about the product price and carbon emission intensity. In particular, the function reflects a price-sensitivity and environmental-sensitivity market where the demand is decreasing with product price increasing and increasing with carbon emission intensity increasing. According to the above descriptions, the demand function can be written as following:

1 $$Q=a-p+\gamma ({e}_{1}+\mu {e}_{2})$$

**Assumption 3**

The production cost generated within a single firm cannot be held privately in full, which is represented by the effect of technological spillovers *θ*, where *θ*_1_ represents the technological externality generated from the manufacturer’s carbon abatement investment, and *θ*_2_ represents the technological externality generated from the supplier’s carbon emissions intensity. Technological spillovers can be interpreted as the fraction of technology that flows between firms (i.e. the higher technological spillover, the higher the frequency of technology flowing between firms), the technology absorption capacity of firms (i.e. the higher technological spillover, the greater the profit from technology flows).

**Assumption 4**

Carbon abatement investment for components and products is conducive to the supplier and the manufacturer changing the old production methods and updating the obsolete machines to reduce the production cost. Thus, based on the conditions of *C*′(*e*_*i*_) > 0 and *C*″(*e*_*i*_) > 0, we consider *C*(*e*_*i*_) is an increasing and convex function for carbon emissions intensity. For convenience, assume the cost of carbon abatement investment for the supplier and the manufacturer both have quadratic mode^24^. So, the cost of carbon abatement investment *C*(*e*_*i*_) can be given as following:

2 $$C({e}_{i})=k\frac{{e}_{i}^{2}}{2}$$

**Assumption 5**

The cost of carbon abatement investment in components and products affect the players’ marginal profit with technological spillovers^25^. And the marginal profit of component and product are given by, respectively.

3 $$\{\begin{array}{c}{y}_{s}^{^{\prime} }=w+{y}_{s}\,\\ {y}_{m}^{^{\prime} }=p+{y}_{m}\end{array}$$

where *y*_*s*_ = *e*_1_ + *θ*_1_ *e*_2_ and *y*_*m*_ = *e*_2_ + *θ*_2_ *e*_1_ are the total effect of carbon abatement investment on contributing to increase the players’ marginal cost^26^, namely those can be seen as the marginal profit of the supplier and manufacturer. In order to simplify the model and facilitate discussion, we assume that the costs of components and products are roughly equal to zero.

### Centralized supply chain

In the centralized supply chain, the decision is made by the supplier and the manufacturer together, namely the manufacturer and the supplier acted as a company or an organization decide the profit of supply chain systems. Based on the above descriptions and assumptions, the profit of supply chain can be obtained as

4 $$\pi =[a-p+\gamma ({e}_{1}+\mu {e}_{2})][p+(1+{\theta }_{2}){e}_{1}+(1+{\theta }_{1}){e}_{2}]-\frac{k{e}_{1}^{2}}{2}-\frac{k{e}_{2}^{2}}{2}$$

Appling the second-order conditions of *π* with respect to *p*, *e*_1_ and *e*_2_, we can get the hessian matrix about the profit of supply chain $$H(p,{e}_{1},{e}_{2})=[\begin{array}{lll}-2 & -1+\gamma -{\theta }_{2} & -1+\gamma \mu -{\theta }_{1}\\ -1+\gamma -{\theta }_{2} & -k+2\gamma (1+{\theta }_{2}) & \gamma (1+{\theta }_{1})+\gamma \mu (1+{\theta }_{2})\\ -1+\gamma \mu -{\theta }_{1} & \gamma (1+{\theta }_{1})+\gamma \mu (1+{\theta }_{2}) & -k+2\gamma \mu (1+{\theta }_{1})\end{array}]$$. We find the determinant is less than zero when the relevant coefficients meet the condition that $2k-{(1+\gamma \mu +{\theta }_{1})}^{2}-{(1+\gamma +{\theta }_{2})}^{2} > 0$. So *H*(*p*, *e*_1_, *e*_2_) is a negative definite for *p*, *e*_1_ and *e*_2_. By solving the first-order conditions of *π*, we have the following proposition in the centralized supply chain.

**Proposition 1** *In the centralized decision, the optimal price and carbon emission intension for the centralized supply chain is as follows*:

$$\{\begin{array}{rcl}{p}^{C} & = & \frac{a[k-(1+{\theta }_{1})(1+\gamma \mu +{\theta }_{1})-(1+{\theta }_{2})(1+\gamma +{\theta }_{2})]}{2k-{(1+\gamma \mu +{\theta }_{1})}^{2}-{(1+\gamma +{\theta }_{2})}^{2}}\\ {e}_{1}^{C} & = & \frac{a(1+\gamma +{\theta }_{2})}{2k-{(1+\gamma \mu +{\theta }_{1})}^{2}-{(1+\gamma +{\theta }_{2})}^{2}}\,\\ {e}_{2}^{C} & = & \frac{a(1+\gamma \mu +{\theta }_{1})}{2k-{(1+\gamma \mu +{\theta }_{1})}^{2}-{(1+\gamma +{\theta }_{2})}^{2}}\,\end{array}$$

Consequently, $({p}^{C},{e}_{1}^{C},{e}_{2}^{C})$ is the optimal decision to maximize the profit of centralized supply chain. Further, the profit of supply chain can be obtained as

5 $${\pi }^{C}=\frac{{a}^{2}k}{2[2k-{(1+\gamma \mu +{\theta }_{1})}^{2}-{(1+\gamma +{\theta }_{2})}^{2}]}$$

**Proposition 2** *In the centralized decision*,

1. $$\frac{\partial {p}^{C}}{\partial k} > 0,\frac{\partial {e}_{1}^{C}}{\partial k} < 0,\frac{\partial {e}_{2}^{C}}{\partial k} < 0;$$
2. $$\frac{\partial {p}^{C}}{\partial \gamma } > 0,\frac{\partial {e}_{1}^{C}}{\partial \gamma } > 0,\frac{\partial {e}_{2}^{C}}{\partial \gamma } > 0;$$
3. $$\frac{\partial {p}^{C}}{\partial {\theta }_{1}} > 0,\frac{\partial {e}_{1}^{C}}{\partial {\theta }_{1}} > 0,\frac{\partial {e}_{2}^{C}}{\partial {\theta }_{1}} > 0;$$
4. $$\frac{\partial {p}^{C}}{\partial {\theta }_{2}} < 0,\frac{\partial {e}_{1}^{C}}{\partial {\theta }_{2}} > 0,\frac{\partial {e}_{2}^{C}}{\partial {\theta }_{2}} > 0;$$
5. $$\{\begin{array}{ll}{e}_{1}^{C} > {e}_{2}^{C}, & if\,{\theta }_{1} < {\theta }_{2}\\ {e}_{1}^{C}={e}_{2}^{C}, & if\,{\theta }_{1}={\theta }_{2}\\ {e}_{1}^{C} < {e}_{2}^{C}, & if\,{\theta }_{1} > {\theta }_{2}\end{array}$$.

Proposition 2 indicates that, with the reduction difficulty coefficient increasing, the product price will increase and carbon emission intensity will decrease. In terms of the effect of technological spillovers and environmental awareness on players’ production cost and market demand, we find that players will increase carbon emission intensity with the technological spillovers and environmental awareness increasing, while the product price will decrease and carbon emission intensity will increase. Further, carbon emission intensity for the supplier is more than that of manufacturer when *θ*_1_ < *θ*_2_; carbon emission intensity for players are equal when *θ*_1_ = *θ*_2_; carbon emission intensity for the supplier is less than that of the manufacturer when *θ*_1_ > *θ*_2_.

### Decentralized supply chain

When the supply chain is decentralized, the supplier and the manufacturer as supply chain players are independent and make their own decisions. In the decentralized supply chain case, using the response function of the manufacturer, the supplier determines the price and carbon emission intensity of components for his profit maximization. Then, given the price and carbon emission intensity of components, the manufacturer decides the price and carbon emission intensity for products so as to maximize his profit^27^. Based on the above descriptions and assumptions, the profit functions of the supplier and manufacturer are respectively represented as

6 $${\pi }_{s}=[a-p+\gamma ({e}_{1}+\mu {e}_{2})](w+{e}_{1}+{\theta }_{1}{e}_{2})-\frac{k{e}_{1}^{2}}{2}$$

7 $${\pi }_{m}=[a-p+\gamma ({e}_{1}+\mu {e}_{2})](p-w+{e}_{2}+{\theta }_{2}{e}_{1})-\frac{k{e}_{2}^{2}}{2}$$

Given *w* and *e*_1_, the second-order conditions of *π*_*m*_ with respect to *p* and *e*_2_, we can get the hessian matrix about the profit of manufacturer $$H(p,{e}_{2})=[\begin{array}{ll}-2 & -1+\gamma \mu \\ -1+\gamma \mu  & -k+2\gamma \mu \end{array}]$$. We find the determinant is greater than zero when the relevant coefficients meet the condition that 2*k* − (1 + *γμ*)^2^ > 0. So *H*(*p*, *e*_2_) is a negative definite and the manufacturer’s profit function is jointly concave in *p* and *e*_2_. Thus, by solving the first-order conditions of *π*_*m*_, the optimal price and carbon emission intensity for product are given as follows

8 $$\bar{p}=\frac{(k-2r\mu )[a+w+{e}_{1}(\gamma -{\theta }_{2})]-(1-\gamma \mu )[a-\gamma \mu w+\gamma {e}_{1}(1+\mu {\theta }_{2})]}{2k-{(1+\gamma \mu )}^{2}}$$

9 $${\bar{e}}_{2}=\frac{(1+\gamma \mu )[a-w+{e}_{1}(\gamma +{\theta }_{2})]}{2k-{(1+\gamma \mu )}^{2}}$$

Anticipating the manufacturer’s best response, the supplier decides *w* and *e*_1_. Substituting $\bar{p}$ and ${\bar{e}}_{2}$ into eq. (6), we take the second-order conditions of *π*_*s*_ with respected *w* and *e*_1_. So the hessian matrix of the supplier profit function is

$$H(w,{e}_{1})=[\begin{array}{ll}-\frac{2k[2k-(1+\gamma \mu )(1+\gamma \mu +{\theta }_{1})]}{{[2k-{(1+\gamma \mu )}^{2}]}^{2}} & \frac{k(\gamma +{\theta }_{2}-1)[2k-{(1+\gamma \mu )}^{2}]-2k{\theta }_{1}(1+\gamma \mu )({\theta }_{2}+\gamma )}{{[2k-{(1+\gamma \mu )}^{2}]}^{2}}\\ \frac{k(\gamma +{\theta }_{2}-1)[2k-{(1+\gamma \mu )}^{2}]-2k{\theta }_{1}(1+\gamma \mu )({\theta }_{2}+\gamma )}{{[2k-{(1+\gamma \mu )}^{2}]}^{2}} & -\frac{k[2k-{(1+\gamma \mu )}^{2}][2(k-\gamma -{\theta }_{2})-{(1+\gamma \mu )}^{2}]-2k{\theta }_{1}(1+\gamma \mu ){(1+\gamma \mu )}^{2}}{{[2k-{(1+\gamma \mu )}^{2}]}^{2}}\end{array}]$$

We find the determinant is greater than zero when the relevant coefficients meet the condition that $4k-2(1+\gamma \mu )(1+\gamma \mu +{\theta }_{1})-{(1+\gamma +{\theta }_{2})}^{2} > 0$. So *H*(*w*, *e*_1_) is a negative definite and the supplier’s profit function is jointly concave in *w* and *e*_1_. Thus, by solving the first-order conditions of *π*_*s*_ with respected *w* and *e*_1_ and substituting the optimal decisions of suppliers into eqs (8 and 9), we have the following proposition in the decentralized supply chain.

**Proposition 3** *In the decentralized decision, the optimal price and carbon emission intensity for the supplier and the manufacturer is as follows*:

$$\{\begin{array}{rcl}{p}^{D} & = & \frac{a[3k-(1+\gamma \mu )(2+\gamma \mu +2{\theta }_{1})-(1+{\theta }_{2})(1+\gamma +2{\theta }_{1})]}{4k-2(1+\gamma \mu )(1+\gamma \mu +{\theta }_{1})-{(1+\gamma +{\theta }_{2})}^{2}}\\ {w}^{D} & = & \frac{a[2k-r-(1+r\mu )(1+r\mu +2{\theta }_{1})-(1+{\theta }_{2})]}{4k-2(1+\gamma \mu )(1+\gamma \mu +{\theta }_{1})-{(1+\gamma +{\theta }_{2})}^{2}}\,\\ {e}_{1}^{D} & = & \frac{a(1+r+{\theta }_{2})}{4k-2(1+\gamma \mu )(1+\gamma \mu +{\theta }_{1})-{(1+\gamma +{\theta }_{2})}^{2}}\,\\ {e}_{2}^{D} & = & \frac{a(1+r\mu )}{4k-2(1+\gamma \mu )(1+\gamma \mu +{\theta }_{1})-{(1+\gamma +{\theta }_{2})}^{2}}\,\end{array}$$

Consequently, $({p}^{D},{w}^{D},{e}_{1}^{D},{e}_{2}^{D})$ is the optimal decision to maximize the profits of decentralized supply chain. Further, the profits of players can be obtained as

10 $${\pi }_{s}^{D}=\frac{{a}^{2}k}{2[4k-2(1+\gamma \mu )(1+\gamma \mu +{\theta }_{1})-{(1+\gamma +{\theta }_{2})}^{2}]}$$

11 $${\pi }_{m}^{D}=\frac{{a}^{2}k[2k-{(1+r\mu )}^{2}]}{2{[4k-2(1+\gamma \mu )(1+\gamma \mu +{\theta }_{1})-{(1+\gamma +{\theta }_{2})}^{2}]}^{2}}$$

**Proposition 4** *In the decentralized supply chain*,

1. $$\frac{\partial {p}^{D}}{\partial k} > 0,\frac{\partial {e}_{1}^{D}}{\partial k} < 0,\frac{\partial {e}_{2}^{D}}{\partial k} < 0;$$
2. $$\frac{\partial {p}^{C}}{\partial \gamma } < 0,\frac{\partial {e}_{1}^{C}}{\partial \gamma } > 0,\frac{\partial {e}_{2}^{C}}{\partial \gamma } > 0;$$
3. $$\frac{\partial {p}^{D}}{\partial {\theta }_{1}} > 0,\frac{\partial {e}_{1}^{D}}{\partial {\theta }_{1}} > 0,\frac{\partial {e}_{2}^{D}}{\partial {\theta }_{1}} > 0;$$
4. $$\frac{\partial {p}^{D}}{\partial {\theta }_{2}} < 0,\frac{\partial {e}_{1}^{D}}{\partial {\theta }_{2}} > 0,\frac{\partial {e}_{2}^{D}}{\partial {\theta }_{2}} > 0;$$
5. $$\{\begin{array}{cc}{e}_{1}^{C} > {e}_{2}^{C}, & if\,{\theta }_{2} > 0\\ {e}_{1}^{C}={e}_{2}^{C}, & if\,{\theta }_{2}=0\end{array}.$$

Proposition 4 indicates that, with reduction difficulty coefficient increasing, the product price will increase and carbon emission intensity will decrease. In terms of the effect of technological spillovers and environmental awareness on players’ marginal cost and market demand, we find that players will increase carbon emission intensity with the technological spillovers and environmental awareness increasing, while the product price will decrease and carbon emission intensity will increase. Further, carbon emission intensity for supplier is more than that of the manufacturer when *θ*_2_ > 0; carbon emission intensity for players are equal when *θ*_2_ = 0.

**Proposition 5** *In comparison of the optimal decisions obtained from the centralized and decentralized supply chain, we hav*e:

$${p}^{C} < {p}^{D};\,{e}_{1}^{C} > {e}_{1}^{D};\,{e}_{2}^{C} > {e}_{2}^{D};\,{\pi }^{C} > {\pi }^{D}$$

Proposition 5 indicates that the price of components in decentralized supply chain is higher than that in centralized supply chain, and carbon emission intensity for the centralized are higher than that of the decentralized. As the supply chain from decentralized to centralized, the supplier and the manufacturer maximize the profits of supply chain together, and reduce the product price to increase demand and profit. Clearly, the reason for higher profitability in the supply chain lies in the fact that any share of carbon emission intensity to prevent “free-riding” behavior. And it has been proved by the researchers that the centralized supply chain performs better than that of the decentralized in terms of profit maximization.

### Supply chain coordination

The above discussion reveals the performance of the supply chain under different scenarios. It is important to provide the contract to coordinate the players, so as to enhance the performance of decentralized. It has been proved by researchers that the centralized supply chain performs better than that of the decentralized in terms of profit maximization or cost minimization^28, 29^. However, it is not always possible for the players to act as a centralized system. Therefore, to ensure better result, the coordination between the players is essential. Combining the revenue-cost sharing contract and Nash bargaining^30^, we propose an effectively coordinated contract, in which the price of component is $w=(1-{t}_{1})(p+{e}_{1}+{e}_{2})+{\theta }_{2}{e}_{1}+{\theta }_{1}{e}_{2}$. In the coordination contract, the sequence of decision-making is the following:

1. The supplier and the manufacturer bargain on the sharing coefficients of revenue and cost respectively given by *t*_1_, *t*_2_. In the bargaining process of coordination contract, the fraction of revenue that the manufacturer obtains is *t*_1_, and the supplier obtains the remaining 1 − *t*_1_ proportion of the revenue. Meanwhile, the cost of carbon emissions reduction is also shared, the fraction of cost that the manufacturer pays is *t*_2_, and the supplier pays the remaining 1 − *t*_2_ proportion of the cost;
2. The supplier decides on carbon emissions intensity *e*_1_ taking the bargaining-coordination contract through revenue-cost sharing and the manufacturer’s reaction function into consideration;
3. The manufacturer decides the product price *p* and carbon emissions intensity *e*_2_ taking the proportion of revenue-cost sharing and carbon abatement investment of supplier into consideration.
4. Therefore, under the given assumption and description, the profits of the supplier and the manufacturer are respectively as follows:

12 $${\pi }_{s}=(1-{t}_{1})[a-p+\gamma ({e}_{1}+\mu {e}_{2})][p+(1+{\theta }_{2}){e}_{1}+(1+{\theta }_{1}){e}_{2}]-(1-{t}_{2})(\frac{k{e}_{1}^{2}}{2}+\frac{k{e}_{2}^{2}}{2})$$

13 $${\pi }_{m}={t}_{1}[a-p+\gamma ({e}_{1}+\mu {e}_{2})][p+(1+{\theta }_{2}){e}_{1}+(1+{\theta }_{1}){e}_{2}]-{t}_{2}(\frac{k{e}_{1}^{2}}{2}+\frac{k{e}_{2}^{2}}{2})$$

We solve the optimal decision for the bargaining-coordination contract through revenue and cost using the backward induction. Hence, given *e*_1_, the second-order conditions of *π*_*m*_ with respect to *p* and *e*_2_, we can get the hessian matrix about the profit function of manufacturer $$H(p,{e}_{2})=[\begin{array}{cc}-2{t}_{1} & -{t}_{1}(1-\gamma \mu +{\theta }_{1})\\ -{t}_{1}(1-\gamma \mu +{\theta }_{1}) & -k{t}_{2}+2\gamma \mu {t}_{1}(1+{\theta }_{1})\end{array}]$$. We find the determinant is greater than zero when the relevant coefficients meet the condition that $2k{t}_{2}-{t}_{1}{(1+r\mu +{\theta }_{1})}^{2} > 0$. Note the Hessian matrix *H*_*B*_ for the profit of bargaining-coordination contract is a negative definite for *p* and *e*_2_. By solving the first-order conditions of *π*_*m*_, the optimal price and carbon emission intensity for the manufacturer are given as follows

14 $$\bar{p}=\frac{k{t}_{2}[a-{e}_{1}(1-\gamma +{\theta }_{2})]-{t}_{1}(1+\gamma \mu +b{\theta }_{1})[a(1+{\theta }_{1})+\gamma {e}_{1}(1-\mu +{\theta }_{1}-\mu {\theta }_{2})]}{2k{t}_{2}-{t}_{1}{(1+\gamma \mu +{\theta }_{1})}^{2}}$$

15 $${\bar{e}}_{2}=\frac{{t}_{1}(1+\gamma \mu +{\theta }_{1})[a+{e}_{1}(1+\gamma +{\theta }_{2})]}{2k{t}_{2}-{t}_{1}{(1+\gamma \mu +{\theta }_{1})}^{2}}$$

Anticipating the manufacturer’s best response, the supplier determines *e*_1_. Substituting $\bar{p}$ and ${\bar{e}}_{2}$ into eq. (12), we take the second-order derivatives of *π*_*s*_ with *e*_1_, and find the profit function of the supplier is strictly concave function. Then, we can obtain carbon emissions intensity for supplier

16 $${e}_{1}^{B}=\frac{a(1+\gamma +{\theta }_{2})[2k{t}_{2}^{2}(1-{t}_{1})-{t}_{1}^{2}(1-{t}_{2}){(1+\gamma \mu +{\theta }_{1})}^{2}]}{{F}_{1}\cdot {t}_{1}^{2}(1-{t}_{2})+{F}_{2}\cdot (1-{t}_{2})-{F}_{3}\cdot 2k{t}_{2}^{2}(1-{t}_{1})}$$

where ${F}_{1}={(1+r+{\theta }_{2})}^{2}{(1+r\mu +{\theta }_{1})}^{2}$, ${F}_{2}={[{t}_{1}{(1+r\mu +{\theta }_{1})}^{2}-2{\rm{k}}{t}_{2}]}^{2}$, ${F}_{3}={(1+r+{\theta }_{2})}^{2}$. Through the coordination mechanism, carbon emissions intensity for the supplier is unique. By comparing ${e}_{1}^{B}$ with carbon emissions intensity in the centralized scenario, we get ${e}_{1}^{C}={e}_{1}^{B}$ if the condition *t*_1_ = *t*_2_ = *t* is satisfied. Then, we obtain that the bargaining-coordination contract can effectively coordinate the supply chain; the profits of players are respectively ${\pi }_{s}^{B}=(1-t)\cdot {\pi }^{C}$ and ${\pi }_{m}^{B}=t\cdot {\pi }^{C}$, where *t*_1_ = *t*_2_ = *t*. So the coefficients *t*_1_ and *t*_2_ are the value negotiated by the bargaining powers.

Only if the supply chain achieve Pareto improvement, namely ${\pi }_{s}^{B}(t)$ and ${\pi }_{m}^{B}(t)$ are not less than the disagreement points ${\pi }_{i}^{D}$(*i* = *s*, *m*), the coordination contract can be accepted by two sides. Then, assuming that *τ* ∈ (0, 1) is the bargaining power of the supplier, while that of the manufacturer is 1 − *τ*. Because the supplier and the manufacturer are respectively the leader and follower, where *τ* ∈ (0.5, 1). Therefore the Nash bargaining of the supply chain is expressed as ${f}_{\tau }:{\rm{\Theta }}\to \Xi$, to each $({\rm{\Omega }},\,{{\pi }_{i}}^{B})\in {\rm{\Theta }}$, ${f}_{\tau }({\rm{\Omega }},{\pi }_{i}^{B})$ is the optimal decision for the bargaining-coordination contract. We now have the Nash bargaining model

17 $$max\theta (t)={[{\pi }_{s}^{B}(t)-{\pi }_{s}^{D}]}^{\tau }{[{\pi }_{m}^{B}(t)-{\pi }_{m}^{D}]}^{1-\tau }$$

where $${\rm{\Omega }}\equiv \{\begin{array}{c}{{\pi }_{s}}^{B}(t),{{\pi }_{m}}^{B}(t)\in \aleph \,\\ {{\pi }_{s}}^{B}(t)\ge {{\pi }_{s}}^{D},\,{{\pi }_{m}}^{B}(t)\ge {{\pi }_{m}}^{D}\end{array}\}$$ and $$\aleph \equiv \{\begin{array}{c}{{\pi }_{s}}^{B}(t),{{\pi }_{m}}^{B}(t):0\le {{\pi }_{i}}^{B}(t)\le {\pi }^{C}\,\\ {{\pi }_{m}}^{B}(t)=g[{{\pi }_{s}}^{B}(t)]={\pi }^{C}-{{\pi }_{s}}^{B}(t)\end{array}\}$$. Based on the theory of Nash bargaining, for bargaining power *τ*, existing $({\rm{\Omega }},{\pi }_{i}^{B})\in {\rm{\Theta }}$ and ${\pi }_{i}^{B}\ge 0$, then the optimal decision of Nash bargaining satisfies the following equation

18 $$g^{\prime} [{\pi }_{s}^{B}(t)]=-\frac{\tau }{1-\tau }\frac{{\pi }_{m}^{B}(t)-{\pi }_{m}^{D}}{{\pi }_{s}^{B}(t)-{\pi }_{s}^{D}}$$

19 $${\pi }_{m}^{B}(t)=g[{\pi }_{s}^{B}(t)]$$

By solving eqs (18 and 19), we can find the optimal decision of the proportion *t* is a function of bargaining power *τ*

$${t}^{\ast }=\frac{(1-\tau )\cdot ({\pi }^{C}-{\pi }_{s}^{D})+\tau \cdot {\pi }_{m}^{D}}{{\pi }^{C}}$$

**Proposition 6** The bargaining-coordination contract through revenue-cost sharing effectively coordinated the decentralized supply chain, we have:

$${p}^{B}={p}^{C};\,{e}_{1}^{B}={e}_{1}^{C};\,{e}_{2}^{B}={e}_{2}^{C};\,{\pi }^{B}={\pi }^{C}$$

Proposition 6 indicates that the bargaining-coordination contract through revenue-cost sharing can improve the performance of the supply chain to the centralized. The supplier and the manufacturer agree the contract which promises that their profits will be higher than the decentralized.

### Numerical study

In this section, a numerical example is presented to obtain the optimal solution, illustrate the effectiveness of above model and the coordination contract. Let the demand potential’s coefficients for products be the same as the numerical example set forth in the literature^31^, the values of coefficients adopted in numerical examples are given as follows, *a* = 50, *γ* = 0.3, *θ*_1_ = 0.6, *θ*_2_ = 0.4, *k* = 20, *μ* = 0.5. Here, the coefficients *a*, *γ* and *μ* represent the number of products. In the demand, we can find that the demand is more sensitive to price than to carbon abatement investment from *γ* < 1, which is reasonable and usually. Carbon abatement investment from the supplier and manufacturer are respectively 60% and 40% reduction in marginal production cost technological spillovers from carbon emission intensity. With the given data, results can be obtained by using software. Based on the theoretical results, the optimal results of supply chain are calculated and shown in Table 2.

**Table 2:** The optimal results under different models.

		*t*	*p*	*w*	*e*_1_	*e*_2_	*π*_s_	*π*_*m*_	*π*
Decentralized scenario		—	36.78	24.35	1.16	0.79	362.92	181.03	543.95
Centralized scenario		—	21.76	—	2.50	2.57	—		734.27
Coordinate contract	*τ* = 0.5	0.38	21.76	19.18	2.50	2.57	455.25	279.02	734.27
	*τ* = 0.6	0.35	21.76	19.98	2.50	2.57	477.28	256.99	734.27
	*τ* = 0.7	0.32	21.76	20.79	2.50	2.57	499.30	234.97	734.27
	*τ* = 0.8	0.30	21.76	21.32	2.50	2.57	513.99	220.28	734.27
	*τ* = 0.9	0.27	21.76	22.13	2.50	2.57	536.02	198.25	734.27
	*τ* = 1	0.25	21.76	22.66	2.50	2.57	553.24	181.03	734.27

From Table 2, some inferences are summarized as follows:

(i) When supply chain is centralized, carbon abatement investment for the supplier and the manufacturer, and supply chain profits are improved. From another point of view, the price of product is lower than that in decentralized supply chain, which means the centralized decision-making can enhance the overall efficiency of the supply chain.;
(ii) The bargaining-coordination contract through revenue-cost sharing has important implications for not only the improvement of supply chain performance but also the coordination between the players of supply chain. The profits of players and supply chain in the bargaining-coordination contract are higher than that of the decentralized scenario, and the prices of component and product are lower than that of the decentralized scenario. The supply chain performance is improved while the incentive compatibility constraint is satisfied through the contract to reach the effectiveness of the centralized and ensure the win–win situation for the players of the supply chain;
(iii) The total profit in the coordinated scenario is constant. That means when the supplier and the manufacturer integrate as a whole system, the change of bargaining power does not affect the profit of supply chain. Whereas, under the impact of technological spillovers, the profit of the supplier increases as the supplier’s bargaining power increases, and the profit of the manufacturer decreases. The outcome of the aforementioned negotiation agendas is unfavorable for the manufacturer, and favorable for the supplier under the equilibrium conditions.

**Figure 1:**
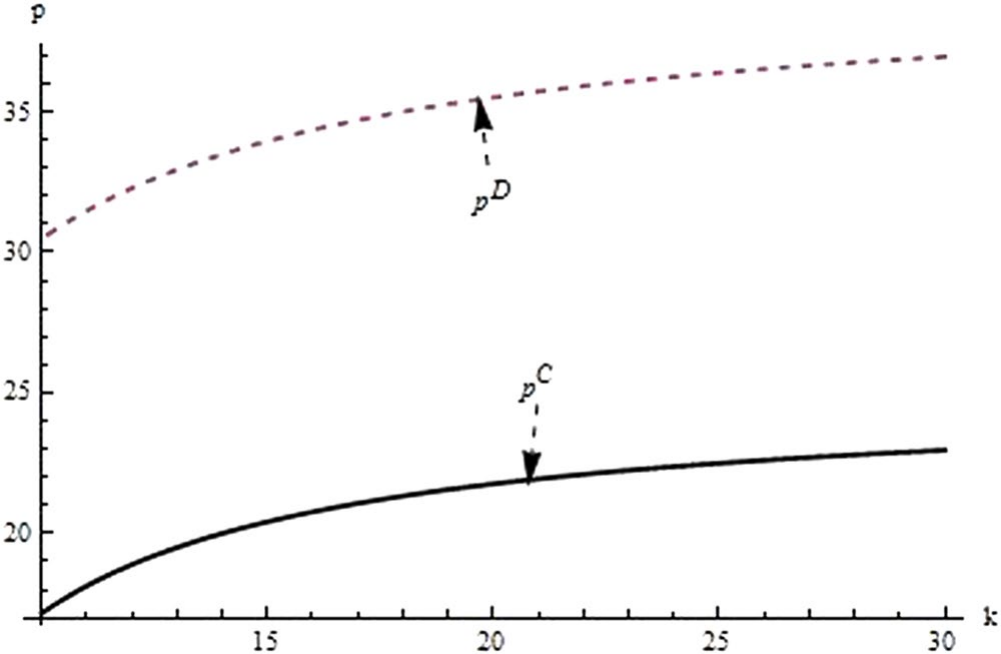
The impact of the reduction difficulty coefficient on the product price.

**Figure 2:**
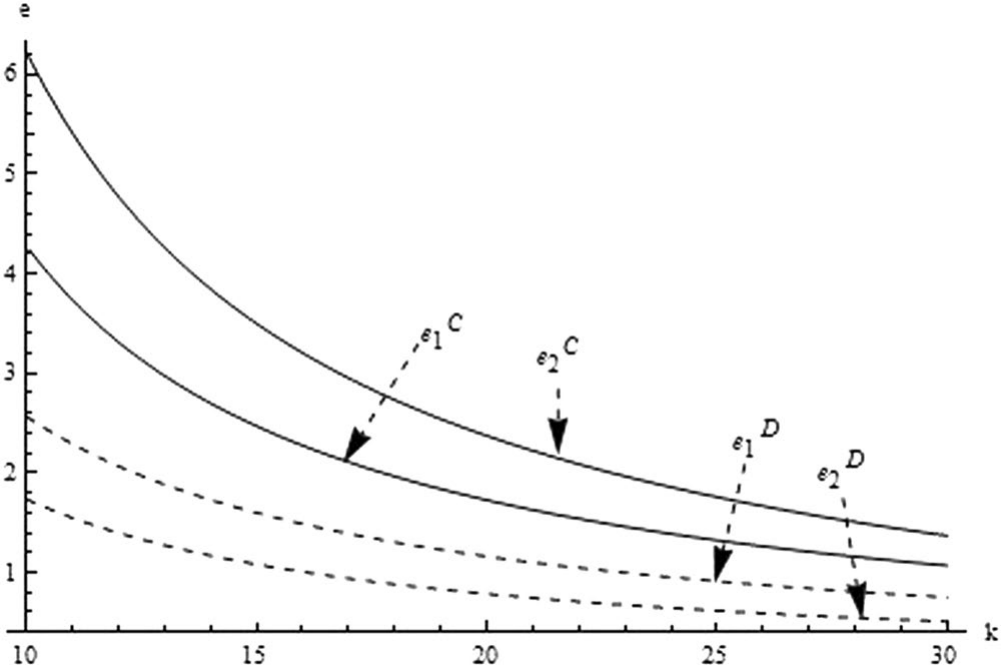
The impact of the reduction difficulty coefficient on carbon emission intensity.

**Figure 3:**
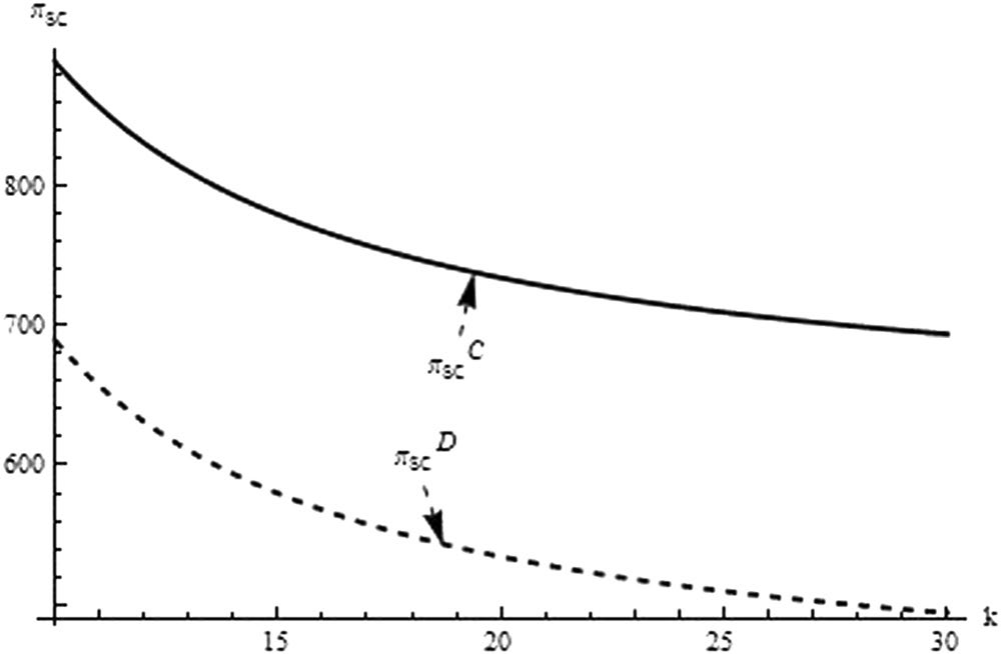
The impact of the reduction difficulty coefficient on the supply chain’s profit.

Then, we firstly analyze and compare the decisions and profits of system and its players in different supply chain structure to investigate the impact of the relevant coefficients (eg. the difficult coefficient of carbon abatement investment, consumer environmental awareness and the technological spillovers) on the optimal decisions and profit. From the Figs 1–3 as the reduction difficulty coefficient varies, the optimal decisions and profits in the different scenarios are respectively illustrated,

(i) The reduction difficulty coefficient has a positive effect on pricing decision in both centralized and decentralized scenario, while a negative effect on carbon emissions intensity and profit. When the players reduce carbon emission in component and product, the greater the reduction difficulty coefficient is, the higher the cost of carbon abatement investment and the lower the profit of supply chain is. Further, the results also show carbon emission intensity and profit is higher for the centralized scenario than the decentralized, product price is lower for the centralized scenario than the decentralized, however the reduction difficulty coefficient changes.

(ii) It is obvious that the reduction difficulty coefficient is a major obstacle of increasing the profits of supply chain in the centralized and decentralized scenarios. To address this issue, the supply chain should give a method to take advantage of the technological spillover. When the reduction difficulty coefficient is great, the supplier as the leader of supply chain, should improve carbon emissions intensity, and guide the manufacturer to increase carbon emissions intensity, to guarantee the profits of players and supply chain increasing.

**Figure 4:**
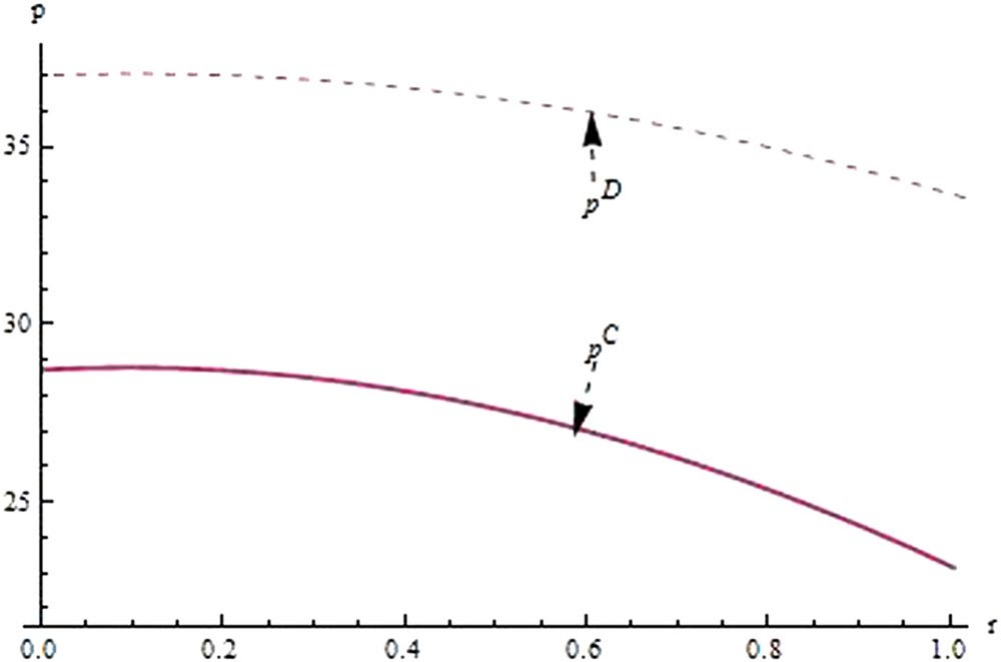
The impact of customers’ environmental awareness on the product price.

**Figure 5:**
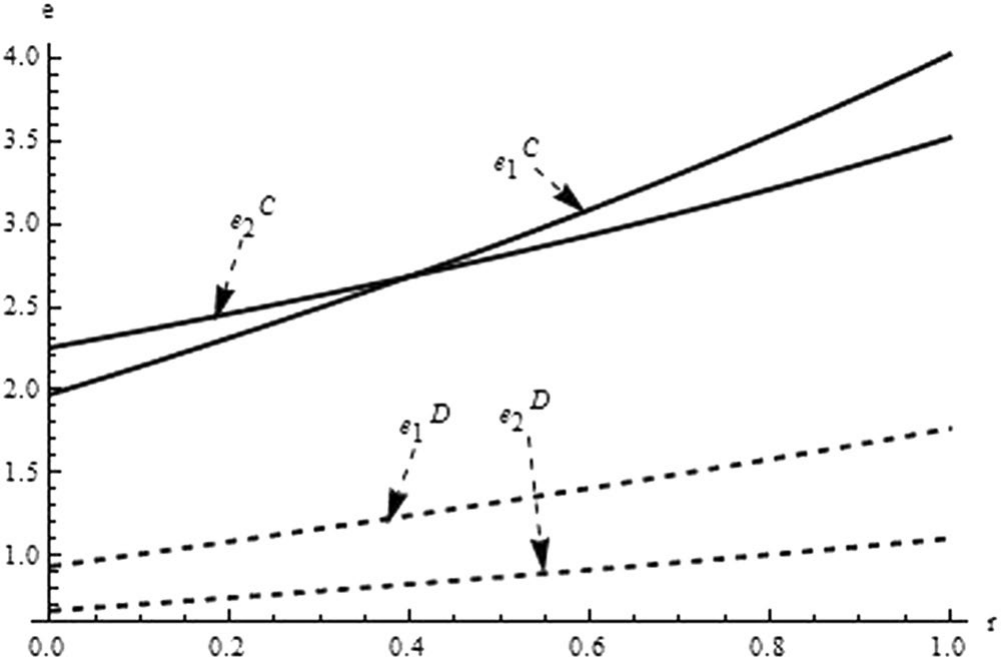
The impact of customers’ environmental awareness on carbon emission intensity.

**Figure 6:**
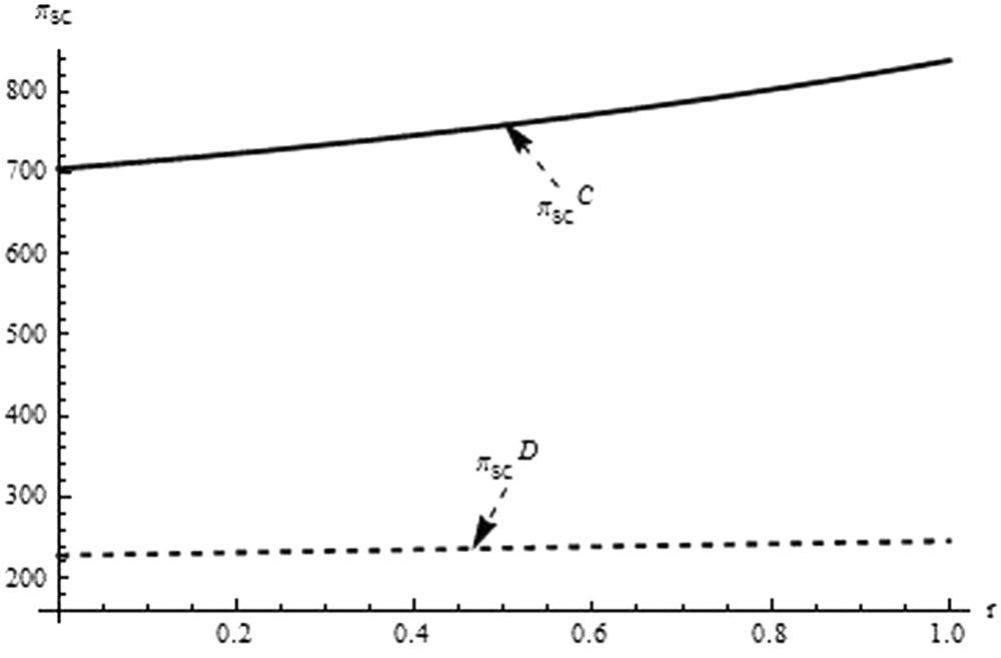
The impact of customers’ environmental awareness on the supply chain’s profit.

From the Figs 4–6 as consumer environmental awareness varies, the optimal decisions and profits in the different scenarios are respectively illustrated,

(i) Customer’s environmental awareness has a negative effect on pricing decisions in both centralized and decentralized scenario, while has a positive effect on the carbon emissions intensity and the profit of supply chain. Clearly, the players in supply chain have the willingness to participate in carbon abatement investment where customers are environmental conscious.
(ii) However, by analyzing the impact of customers’ environmental awareness on carbon emission intensity for the supplier and manufacturer, we obtain some interesting results. Although the trends that carbon emission intensity increases with customers’ environmental awareness in different scenarios are the same, those for the supplier and manufacturer are different. In the decentralized scenario, when customers; environmental awareness reaches a certain threshold, the results of carbon emission intensity for the supplier and manufacturer are opposites. But in the centralized scenario, the supplier offers a higher degree of carbon emission intensity than that offered by the manufacturer. The reason in appearance of this phenomenon is that the establishment of Stackelberg game decides the government to play a leader role based on environmental awareness and “free-ride” behavior.

**Figure 7:**
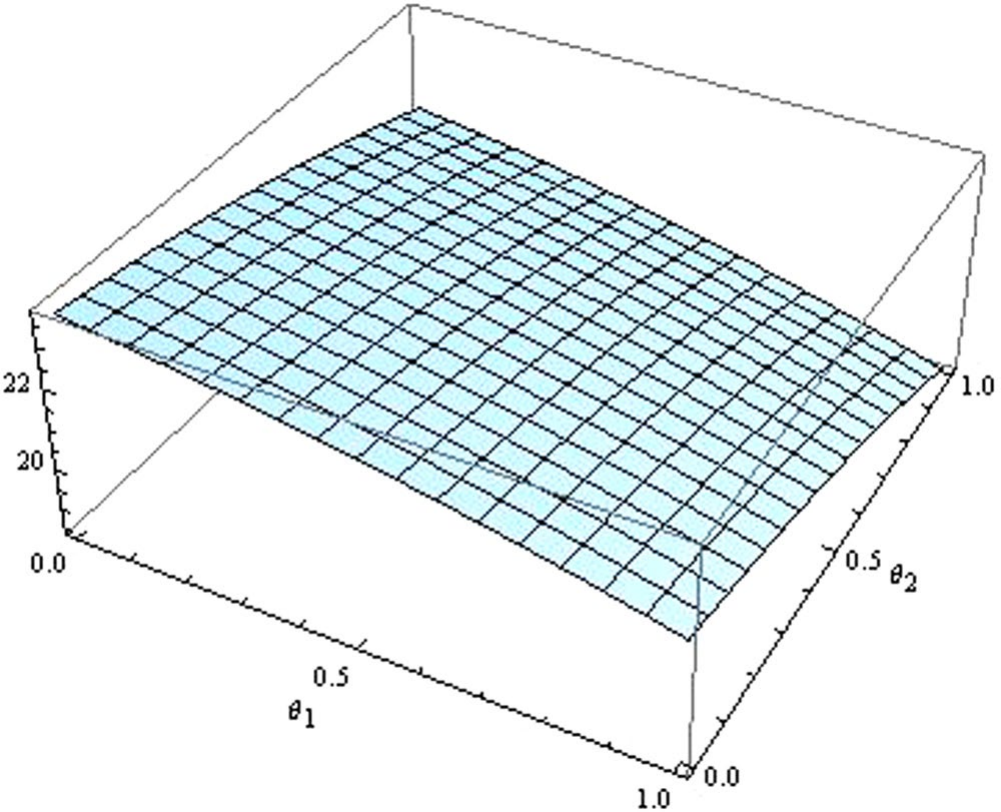
The impact of technological spillovers on the product price in the centralized scenario.

**Figure 8:**
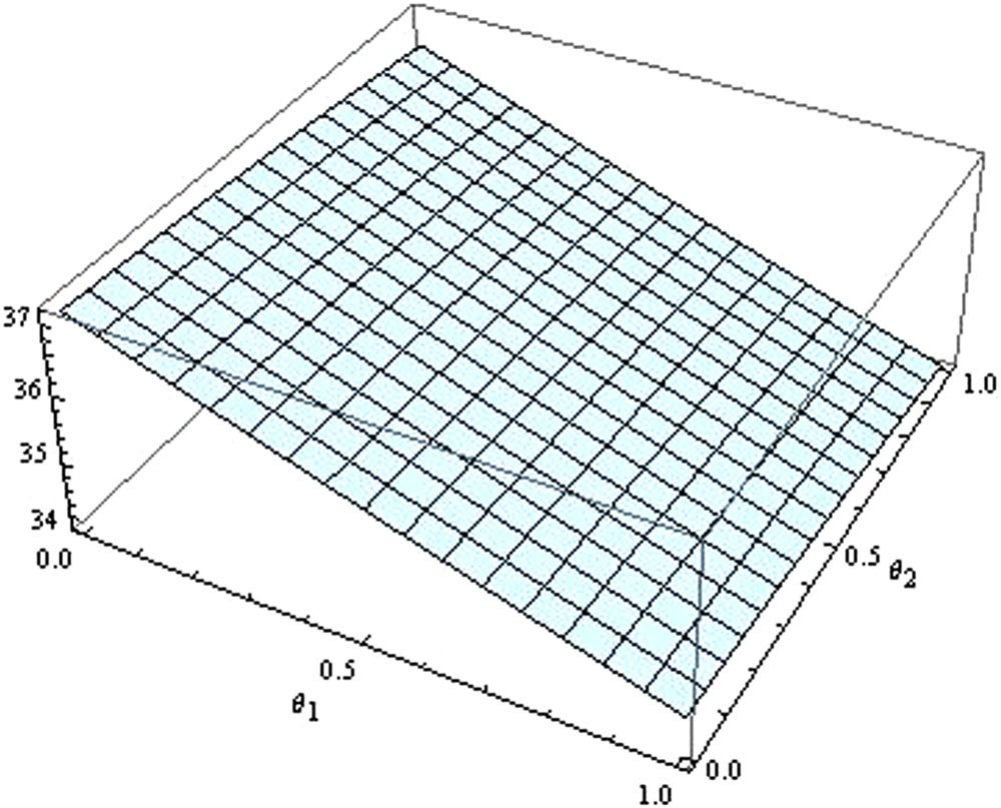
The impact of technological spillovers on the product price in the decentralized scenario.

**Figure 9:**
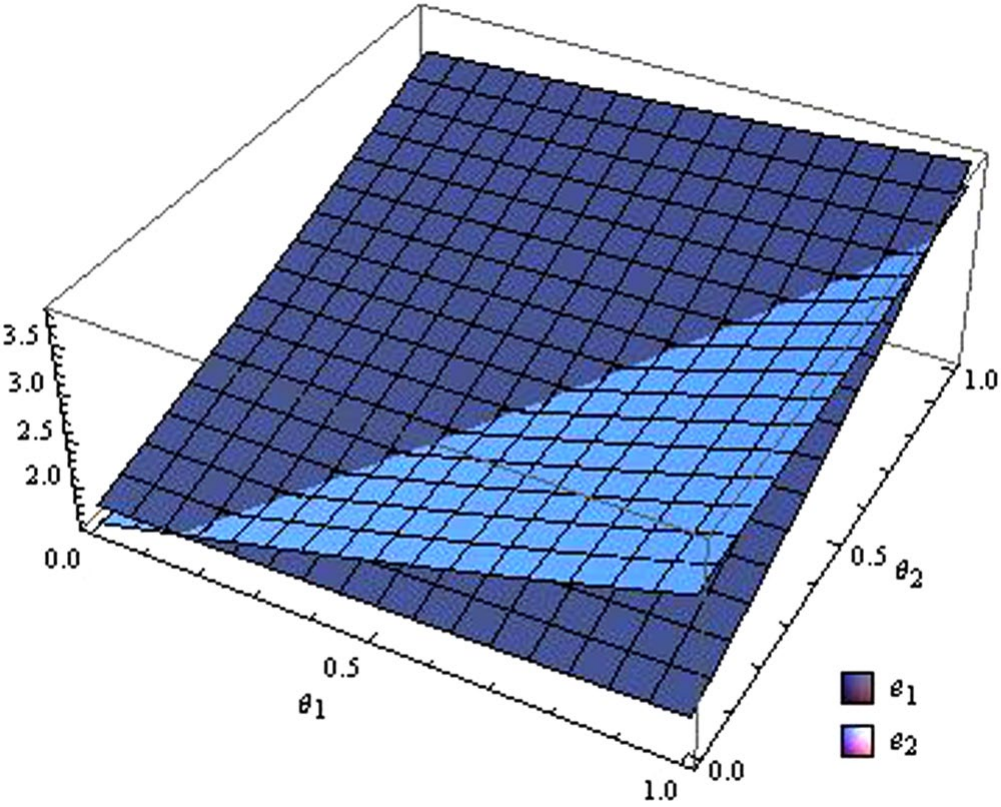
The impact of technological spillovers on carbon emissions reduction in the centralized scenario.

**Figure 10:**
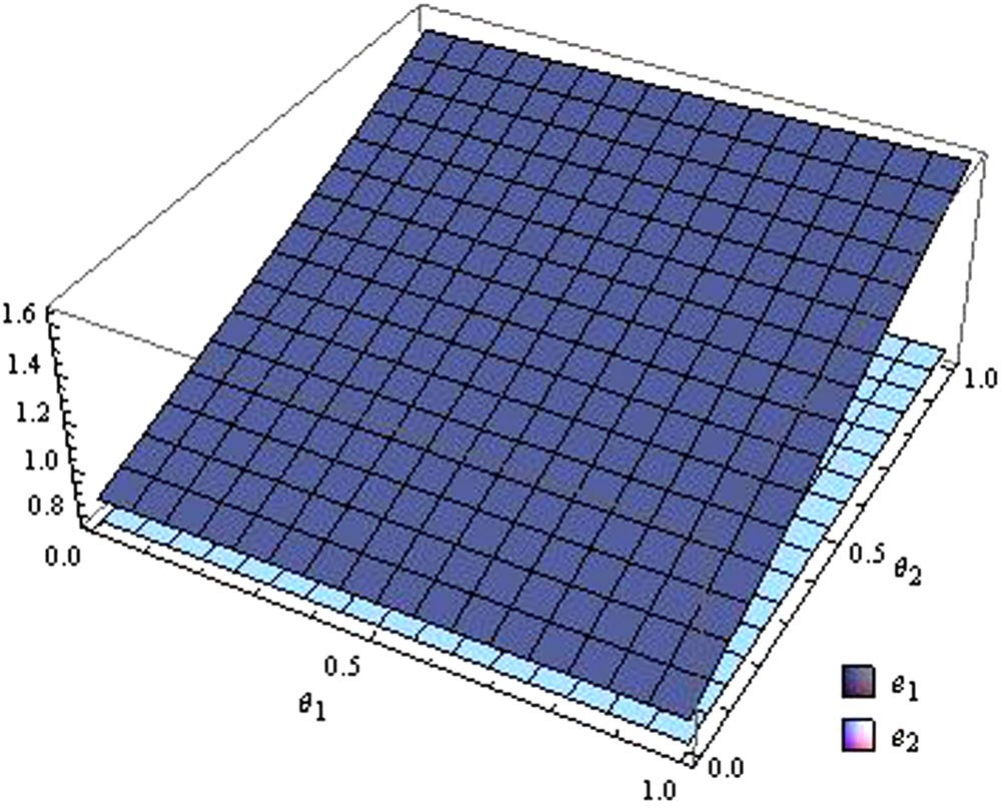
The impact of technological spillovers on carbon emissions reduction in the decentralized scenario.

**Figure 11:**
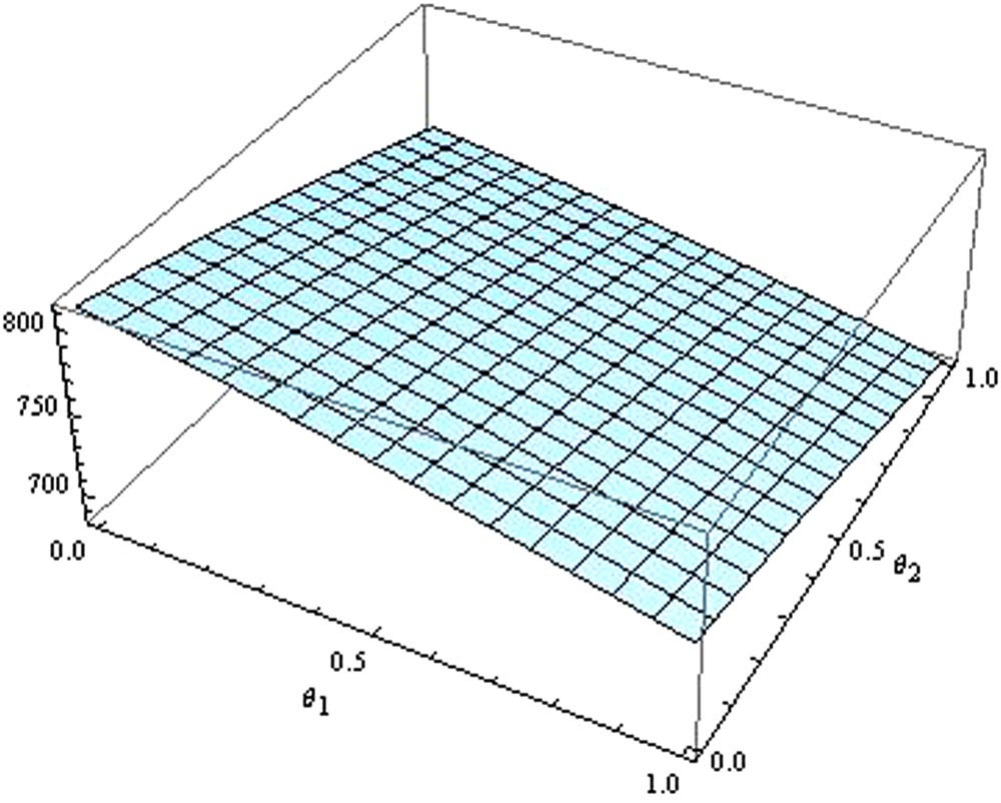
The impact of technological spillovers on the supply chain’s profit in the centralized scenario.

**Figure 12:**
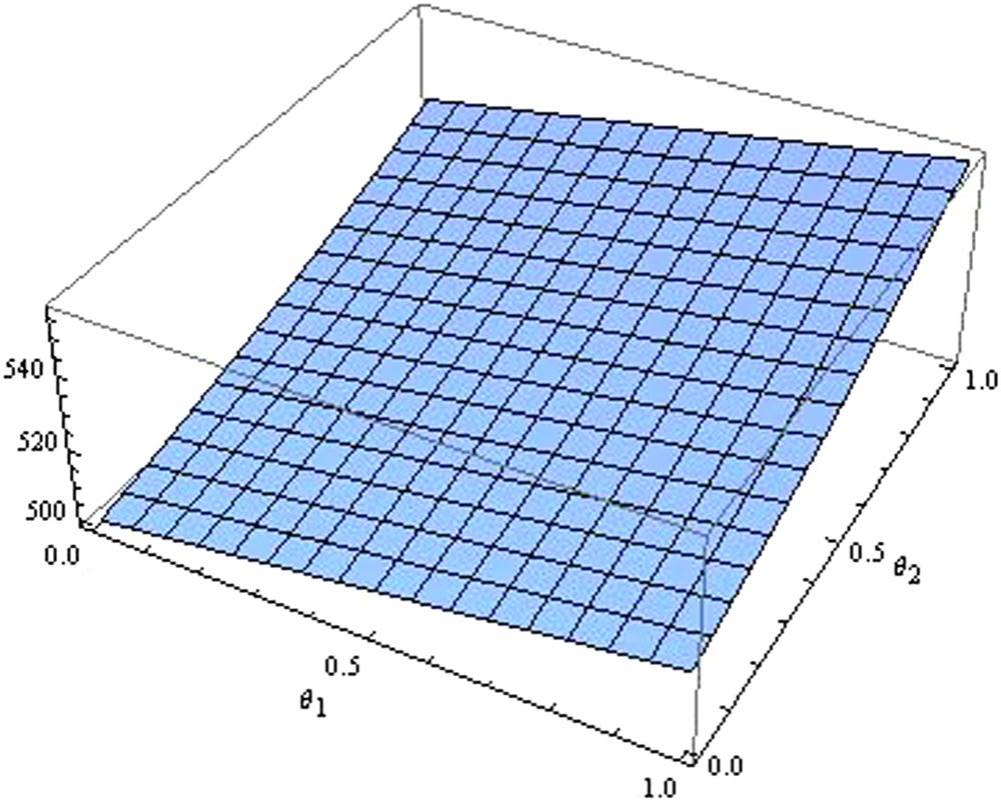
The impact of technological spillovers on the supply chain’s profit in the decentralized scenario.

From the Figs 7–12 as the coefficients of technological spillovers varies, the optimal decisions and profits in different scenarios are respectively illustrated:

(i) The technological spillovers of carbon emissions intensity have a positive effect on the carbon abatement investment for the players, and a negative effect on the pricing in the decentralized and centralized scenarios. When the technological spillovers of carbon emissions intensity are low, the production cost is high, the cost of carbon abatement investment is also high and the price of products will be set higher. When the technological spillovers of carbon emissions intensity are high, as the production cost decreasing, the manufacturer will take advantage of the consumer’s proactive response to reduce the price of product, increase carbon abatement investment of the supplier and the manufacturer.
(ii) Comparing the centralized and decentralized scenarios, it is shown from in Figs 7 and 8 that the price change interval in the centralized are greater than that in the decentralized with the technological spillovers increase. The similar situation also holds in carbon abatement investment and the profit of supply chain. It can be shown from Figs 9 and 10 that, in centralized supply chain, the impact of *θ*_1_ and *θ*_2_ on supply chain is obvious. The reason is that carbon abatement investment is made by the supplier and the manufacturer together to maximize the supply chain profit considering the effect of technological spillovers. Moreover, the production cost of players in supply chain is reduced with the increase of technological spillovers, the product price is also decreased, and accordingly the product demand is increased.
(iii) Comparing the centralized and decentralized scenarios, it can be shown from Figs 9 and 10 that, in the decentralized supply chain, the impact of *θ*_1_ on supply chain carbon emissions reduction is not obvious and the impact of *θ*_2_ on carbon emissions intensity is obvious. This indicates that carbon abatement investment of supplier is obvious, and that of manufacturer is not obvious. The reason is that the supplier acted as the leader of supply chain usually induce manufacturer to increase carbon emissions reduction by increase own carbon abatement investment. However, the manufacturer considers the carbon abatement investment from the aspect of own profit, and this will lead to the “free rider” behavior of manufacturer by employing carbon abatement investment for supplier. And technological spillovers between the supplier and the manufacturer amplify the impact of “free-ride” behavior, the supplier always have incentives to reduce carbon emission, but the cooperation of players will achieve as well as improve the profits only when technological spillovers are large.

## Conclusion

Innovation is the primary measure to maintain the advance in the competition, nevertheless the technological spillover and environmental awareness are easy to play an inhibitory effect. While, this is the first study from the perspective of the two-echelon supply chain consisting of one-single supplier and one-single manufacture, both of whom conduct carbon emissions reduction with vertical technological spillover and environmental awareness to reduce their production costs and improve their profits. Using the method of Stackelberg game, we can obtain the optimal decisions and coordinate supply chain by the bargaining contract through revenue-cost sharing. In our work, we get several findings: First, whether it is the centralized or decentralized scenarios, the profit and carbon emissions intensity for players always increase with the technological spillovers or environmental awareness increasing, conversely the product price decreases. Moreover, with the reduction difficulty coefficient increasing, carbon emissions intensity will decrease, and the players will take various protective measures to the technologies, when the technological spillovers are relatively small. Further, the bargaining-coordination contract through revenue-cost sharing can enhances the performance of decentralized supply chain perfectly to reach that of centralized supply chain, without harming players’ profits. It is beneficial for the supplier and manufacturer, and the proportions of revenue-cost allocation depend on the bargaining powers of them.
